# The Relationship between Active Trachoma and Ocular *Chlamydia trachomatis* Infection before and after Mass Antibiotic Treatment

**DOI:** 10.1371/journal.pntd.0005080

**Published:** 2016-10-26

**Authors:** Athumani M. Ramadhani, Tamsyn Derrick, David Macleod, Martin J. Holland, Matthew J. Burton

**Affiliations:** 1 Clinical Research Department, Faculty of Infectious and Tropical Diseases, London School of Hygiene & Tropical Medicine, United Kingdom; 2 Kilimanjaro Christian Medical Centre, Moshi, Tanzania; 3 Tropical Epidemiology Group. London School of Hygiene & Tropical Medicine, United Kingdom; 4 Moorfields Eye Hospital, London, United Kingdom; University of California San Diego School of Medicine, UNITED STATES

## Abstract

**Background:**

Trachoma is a blinding disease, initiated in early childhood by repeated conjunctival infection with the obligate intracellular bacterium *Chlamydia trachomatis*. The population prevalence of the clinical signs of active trachoma; ‘‘follicular conjunctivitis” (TF) and/or ‘‘intense papillary inflammation” (TI), guide programmatic decisions regarding the initiation and cessation of mass drug administration (MDA). However, the persistence of TF following resolution of infection at both the individual and population level raises concerns over the suitability of this clinical sign as a marker for *C*. *trachomatis* infection.

**Methodology/Principle Findings:**

We systematically reviewed the literature for population-based studies and those including randomly selected individuals, which reported the prevalence of the clinical signs of active trachoma and ocular *C*. *trachomatis* infection by nucleic acid amplification test. We performed a meta-analysis to assess the relationship between active trachoma and *C*. *trachomatis* infection before and after MDA. TF and *C*. *trachomatis* infection were strongly correlated prior to MDA (r = 0.92, 95%CI 0.83 to 0.96, p<0.0001); the relationship was similar when the analysis was limited to children. A moderate correlation was found between TI and prevalence of infection. Following MDA, the relationship between TF and infection prevalence was weaker (r = 0.60, 95%CI 0.25 to 0.81, p = 0.003) and there was no correlation between TI and *C*. *trachomatis* infection.

**Conclusions/Significance:**

Prior to MDA, TF is a good indicator of the community prevalence of *C*. *trachomatis* infection. Following MDA, the prevalence of TF tends to overestimate the underlying infection prevalence. In order to prevent unnecessary additional rounds of MDA and to accurately ascertain when elimination goals have been reached, a cost-effective test for *C*. *trachomatis* that can be administered in low-resource settings remains desirable.

## Author Summary

Trachoma is the leading infectious cause of blindness worldwide, caused by the bacterium *Chlamydia trachomatis*. Repeated infection of the conjunctiva during childhood can initiate chronic conjunctival inflammation. This can lead to conjunctival scarring, in turning of the eyelashes, abrasion of the eyelashes on the cornea and eventually blindness later in adulthood. The World Health Organization recommends mass drug administration (MDA) for infection control when the prevalence of the clinical sign of Active Trachoma (TF) is ≥10% in 1–9 year olds. This systematic review of the literature examined the relationship between TF and *C*. *trachomatis* infection before and after MDA in order to evaluate the usefulness of TF for guiding trachoma control programmes. The population prevalence of TF and *C*. *trachomatis* infection were strongly correlated prior to MDA, however the relationship was weaker after MDA with a greater tendency for TF to overestimate the underlying infection prevalence. A cost effective test for *C*. *trachomatis* suitable for use in low resource settings could prevent unnecessary additional rounds of MDA in the population and could identify when trachoma elimination goals have been reached at an earlier time point.

## Introduction

**Fig 1:**
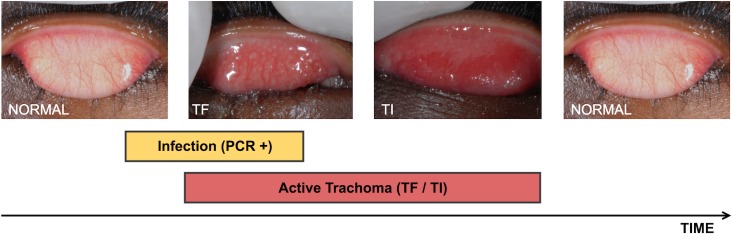
The natural history of an episode of ocular *C*. *trachomatis* infection and the associated conjunctival inflammatory response. Trachomatous Inflammation–Follicular (TF): the presence of five or more follicles in the upper tarsal conjunctiva. Trachomatous Inflammation–Intense (TI): pronounced inflammatory thickening of the upper tarsal conjunctiva that obscures more than half of the normal deep tarsal vessels [2].

Sight loss from trachoma is the end result of a scarring disease process that is initiated in early childhood by the obligate intracellular bacterium *Chlamydia trachomatis* [1]. Repeated infection of the conjunctiva by *C*. *trachomatis* causes a recurrent chronic follicular conjunctivitis (TF) of the upper eyelid mucosal surface (Fig 1) [2]. This can sometimes be particularly severe with intense papillary inflammation (TI). Together, TF and / or TI are collectively referred to as “Active Trachoma”. Conjunctival scarring gradually develops, usually becoming visible from early adulthood. Eventually the scarring causes the eyelashes to turn in and scratch the surface of the eye. If left uncorrected, trichiasis traumatises the cornea surface, resulting in opacification and sight loss.

There is a variable relationship between the clinical signs of Active Trachoma and the presence of *C*. *trachomatis* infection. The natural history of an infection episode in children is probably characterised by a brief “pre-clinical” phase, in which there is detectable infection but the clinical signs of the inflammatory response are yet to develop (Fig 1). Human volunteer experiments in which the conjunctiva was inoculated with *C*. *trachomatis* indicated that the signs of disease typically take about 10 days to develop in previously uninfected individuals [3]. This is followed by a variable period of time in which both infection and disease can be detected at the same time; this may last for several days to many weeks. The immune response brings the infection under control, completely clearing it or reducing it to undetectable levels. However, inflammatory clinical signs persist, typically lasting several weeks after the resolution of the infection. In children aged 4–15 years, data from longitudinal cohort studies estimate the median duration of infection range between 23 days and 8 weeks, and the median duration of disease between 54 days and 18 weeks [4–6]. The duration of disease and infection declines with increasing age. Therefore, at an individual level there is frequently a large mismatch between when infection can be detected and the clinical signs of disease are found [7].

The most recent estimates from the World Health Organization (WHO) Alliance for the Global Elimination of Trachoma by 2020 (GET2020) estimates indicate that about 200 million people live in trachoma endemic areas in 42 countries, 2.2 million have visual impairment or blindness, and about 3.2 million have trichiasis [8]. To meet this large public health challenge, the GET2020 Alliance recommends the implementation of the SAFE Strategy which tackles the disease at different stages: **S**urgery to correct trichiasis, **A**ntibiotics to treat chlamydial infection and **F**acial cleanliness and **E**nvironmental improvements to suppress transmission of the infection [1].

The antibiotic azithromycin is being used in community-wide mass drug administration (MDA); it is given as a single oral dose on an annual basis in endemic districts. Decisions around when to initiate and stop MDA are guided by the prevalence of TF in children in endemic communities. According to the current guidelines, azithromycin MDA is indicated for entire districts where the prevalence of TF in 1–9 year olds is ≥10%. Moreover, the determination of whether a program has controlled the active stage of trachoma as a public health problem also rests on the district level prevalence of TF [9].

Therefore, in view of the significance attached to TF for making programmatic decisions, it is important to understand the relationship between active disease and chlamydial infection in endemic communities. It is probable that this relationship changes after the introduction of MDA and will vary with different levels of endemicity. Several studies have specifically investigated what clinical signs can tell us about infection [7, 10–19]. However, in addition there are many other population-based studies, which report on both disease and infection, that can contribute information. Here we systematically review the published literature for reports that can inform our understanding of the relationship between clinical signs of active disease and *C*. *trachomatis* infection, both before and after the introduction of MDA.

## Methods

In this review we included population-based studies and studies involving a random selection of participants that report the relationship between signs of Active Trachoma (TF, TI, TF/TI) and the detection of ocular *C*. *trachomatis* by nucleic acid amplification tests (NAAT), including polymerase chain reaction (PCR), polymerase chain reaction/enzyme immunoassay (PCR-EIA), ligase chain reaction (LCR) and transcription-mediated amplification (TMA). We excluded studies which did not test individuals without Active Trachoma for infection. References were identified through searching PubMed for articles using the terms (i) “trachoma” AND “*Chlamydia trachomatis*”, (ii) “Trachoma” AND “PCR”, (iii) “Trachoma” AND “LCR”, (iv) “Trachoma” AND “azithromycin”. The search was limited to 1991 onwards, the year of the first report of the use of PCR to test for ocular *C*. *trachomatis* infection in a trachoma endemic population [20]. The search was last updated on 5^th^ May 2016.

The titles and abstracts of all articles resulting from these searches were screened by two authors (AMR, MJB) for potentially eligible publications. These two authors then independently assessed the potentially eligible articles for inclusion and extracted the data. The bibliographies of publications meeting the inclusion criteria were also reviewed for any additional publications not already identified. Studies were excluded where the participant sample was not population-based. Several studies were identified with multiple related published reports arising from the same data. These are considered as a single study, but for completeness we include all the relevant references.

Core information was extracted using a standardised form. The core information included country, year of publication, study design, study population size, age group, TF prevalence, TI prevalence, TF/TI prevalence (if TF alone was not reported), *C*. *trachomatis* infection prevalence, diagnostic test used, grading system, use of antibiotic for infection control. The clinical grading system was recorded: the 1981 Detailed WHO FPC System or the 1987 Simplified WHO Trachoma Grading System [2, 21]. For the purpose of this review the prevalence levels of TF in the populations studied were categorised as follows: Hypoendemic <10%, Mesoendemic 10–20% and Hyperendemic >20% [12].

Due to the heterogeneity in the study designs and the reporting of data, only a limited meta-analysis was performed. Studies report the results for different age groups. Where available we separately present both the “All Age” results and those for children (≤15 years). In the meta-analysis we use the results from children only where these are available; where these are not available, the all age results are used. Summary graphs are presented of the relationship between: (i) the community prevalence of disease signs and *C*. *trachomatis* infection, (ii) the sensitivity, specificity, positive predictive value (PPV), negative predictive value (NPV) of TF for infection, by the community prevalence of disease signs. The degree of correlation between these was tested using Pearson’s correlation coefficient, weighted by study size. The relationship between signs and infection was analysed separately for pre-treatment and post-treatment data, then a test for interaction was performed on the two datasets combined to test whether the association between signs and infection was similar in the pre and post-treatment data. Forest plots were generated to illustrate the strength and heterogeneity of the relationship between disease signs and detection of infection; study heterogeneity was estimated using the I^2^ statistic. Hierarchical summary receiver operating characteristics (HSROC) curves were plotted to illustrate the relationship between the sensitivity and specificity of signs of TF for the presence of *C*. *trachomatis* infection and a pooled estimate of sensitivity and specificity was made in both the pre and the post MDA studies. All analyses were performed using Stata 13. A Preferred Reporting Items for Systematic Reviews and Meta-Analyses (PRISMA) checklist and flow diagram are included in the supporting information (S1 Checklist and S1 Fig).

## Results

The search returned a list of 718 publications. The findings from pre and post-MDA studies are presented separately. Thirty-six separate population-based studies reported findings prior to the introduction of MDA. Several had multiple related publications; yielding a total of 48 publications (Table 1). Twenty-one studies reported findings following the introduction of MDA met inclusion criteria, in a total of 35 publications (Table 2).

**Table 1:** Active Trachoma and *Chlamydia trachomatis* (Ct) infection prior to mass antibiotic treatment.

Country, Year, Ref.	Study design	Participants	Active Trachoma %	Ct %	Ct+/TF+	Ct+/TF-	Comments
Tanzania, 1991, [20]	Baseline cross-sectional population-based data for a treatment trial. One child aged 1–7 years was randomly selected from 234 households in a village.	1–7 years: 234	1–7 years: ● TF/TI 58.5% (137/234 ● TI 15.8% (37/234)	1–7 years: 47.9% (112/234)	1–7 years: 65.0% (89/137)	1–7 years: 23.7% (23/97)	● Hyperendemic setting ● Clinical grading: simplified WHO system ● Chlamydia test: in-house PCR-EIA for *OMP1*.
Gambia, 1994, [29]	Cross-section survey of the entire population of two villages	● All ages: 1332 ● 0–15 years: 714	All ages: ● TF/TI 15.0% (200/1332)0–15 years: ● TF/TI 29.8% (183/714)	● All ages: 17.2% (229/1332) ● 0–15 years: 29.8% (183/714)	● All ages: 72.0% (144/200) ● 0–15 years: 73.2%% (134/183)	● All ages: 7.5% (85/1132) ● 0–15 years: 9.2% (49/531)	● Mesoendemic setting ● Clinical grading: detailed WHO-FPC system ● Chlamydia test: in-house PCR for plasmid, detected by agarose gel electrophoresis.
Gambia, 1994, [22]	Cross-section survey of the entire population of one village. Only 133 individuals were tested for Ct infection by PCR. All active cases were tested. A sample of 37 normal individuals from two households were tested.	All ages: 844	All ages: ● TF/TI 11.4% (96/844)	Data not available	All ages: 51.0% (49/96)	All ages: 5.4% (2/37)	● Mesoendemic setting ● Clinical grading: detailed WHO-FPC system ● Chlamydia test: in-house PCR for plasmid, detected by agarose gel electrophoresis. ● The clinically normal group are not necessarily representative of the whole community as they are drawn from two households only.
Nepal, 1998, [30]	All children under 11 years in primary school and all children from four randomly selected households.	0–10 years: 70	0–10 years: ● TF/TI 38.6% (27/70) ● TI 7.1% (5/70)	0–10 years: 57.1% (40/70)	0–10 years: 66.7% (18/27)	0–10 years: 51.2% (22/43)	● Hyperendemic setting ● Clinical grading: simplified WHO system ● Chlamydia test: in-house PCR for *OMP1*. ● The sample was not necessarily representative of the child population of this community.
Egypt, 1999, [10, 31]	Baseline cross-sectional, population-based survey; pre-treatment data from a RCT of azithromycin vs tetracycline.	● All ages: 2069 ● 1–10 years: 731	All ages: ● TF/TI 19.7% (408/2069) ● TI 5.8% (120/2069) ● 1–10 years:TF/TI 48.3% (353/731) ● TI 13.0% (95/731)	● All ages: 35.7% (739/2069) ● 1–10 years: 48.6% (355/731)	● All ages: 66.9% (273/408) ● 1–10 years: 69.4% (245/353)	● All ages: 31.6% (466/1661) ● 1–10 years: 29.1% (110/378)	● Hyperendemic setting ● Clinical grading: detailed WHO-FPC system ● Chlamydia test: LCR, Abbott Laboratories.
Gambia, 1999, [31]	Baseline cross-sectional, population-based survey; pre-treatment data from a RCT of azithromycin vs tetracycline.	● All ages: 1747 ● 0–10 years: 730	All ages: ● TF/TI 15.8% (277/1747) ● TI 7.0% (122/1747)0–10 years: ● TF/TI 33.8% (247/730) ● TI 13.7% (100*/730)	● All ages: 35.9% (628/1747) ● 0–10 years: 39.3% (287/730)	All ages: 59.9% (166/277)	All ages: 31.4% (462/1470)	● Hyperendemic setting ● Clinical grading: detailed WHO-FPC system ● Chlamydia test: LCR, Abbott Laboratories. ● Main results presented for all ages, possible to derive some of the indicators for the 0–10 year group.
Tanzania, 1999, [31]	Baseline cross-sectional, population-based pre-treatment survey; data from a RCT of azithromycin vs tetracycline.	● All ages: 2653 ● 0–10 years: 940	All ages: ● TF/TI 31.8% (844/2653) ● TI 16.4% (436/2653) 0–10 years: ● TF/TI 60.1% (565/940) ● TI 24.7% (233*/940)	● All ages: 18.5% (491/2653) ● 0–10 years: 34.6% (325/940)	All ages: 48.3% (408/844)	All ages: 4.6% (83/1809)	● Hyperendemic setting ● Clinical grading: detailed WHO-FPC system ● Chlamydia test: LCR, Abbott Laboratories. ● Main results presented for all ages, possible to derive some of the indicators for the 0–10 year group. ● In this study there was some concern about sample degradation, leading to an underestimate of infection
Nepal, 1999, [11]	Cross-sectional survey of all children in six villages. Swabs and LCR test done on all active cases and 1/8 without active disease.	0–10 years: 726	years: ● TF/TI 6.3% (46/726)	0–10 years: 0%(0/90)	0–10 years: 0%(0/46)	0–10 years: 0% (0/44)	● Hypoendemic setting ● Clinical grading: simplified WHO system ● Chlamydia test: LCR, Abbott Laboratories.
Nepal, 2001, [23]	Baseline cross-sectional population-based data from a RCT of mass vs targeted antibiotic treatment. Children recruited from 17 wards. Only the children with active disease and a similar sized random sample of the others were tested by LCR.	1–7 years: 619	1–7 years: ● TF/TI 19.0% (118/619)	Data not available	1–7 years: 24.8% (29/117)	1–7 years: 4.2% (5/118)	● Mesoendemic setting ● Clinical grading: simplified WHO system ● Chlamydia test: LCR, Abbott Laboratories.
Gambia, 2003, [7, 27, 32, 33]	Baseline population-based survey of a longitudinal study of MDA. All residents of 14 villages.	● All ages: 1319 ● 1–9 years: 492	All ages: ● TF/TI 7.8% (103/1319) ● TI 1.4% 19/13191–9 years: ● TF 16.3% (80/492) ● TI 2.6% (13/492)	● All ages: 7.2% (95/1319) ● 1–9 years: 9.3% (46/492)	● All ages: 22.3% (23/103) ● 1–9 years: 23.8% (19/80)	● All ages: 5.9% (72/1216) ● 1–9 years: 6.6% (27/412)	● Mesoendemic setting ● Clinical grading: detailed WHO-FPC system ● Chlamydia test: Amplicor PCR, Roche.
Tanzania, 2003, [27, 34, 35]	Baseline population-based survey for a longitudinal study of MDA. All residents of a single sub-village.	All ages: 956	All ages: ● TF/TI 18.2% (174/956) ● TI 11.6% (111/956)	All ages: 9.5% (91/956)	All ages: 33.3% (58/174)	All ages: 4.2% (33/782)	● Hyperendemic Setting ● Clinical grading: simplified WHO system ● Chlamydia test: Amplicor PCR, Roche
Tanzania, 2003, [27, 36–39]	Baseline population-based survey for a longitudinal study of MDA. All residents of a single village.	● All ages: 871 ● 0–10 Years: 325	All ages: ● TF/TI 35.8% (312/871) ● TI 10.9% (95/871) ● 0–10 Years:F/TI 72.6% (236*/325)	● All ages: 56.9% (496/871) ● 0–10 Years: 64.9% (211*/325)	All ages: 77.2% (241/312)	All ages: 45.6% (255/559)	● Hyperendemic setting ● Clinical grading: simplified WHO system ● Chlamydia test: Amplicor PCR, Roche
Ethiopia, 2004, [17]	Random selection of 100 children aged 1–6 years from four villages.	1–6 years: 100	1–6 years: ● TF 79% (79/100) ● TI 55% (55/100)	1–6 years: 63.0% (63/100)	1–6 years: 72.2% (57/79)	1–6 years: 28.6% (6/21)	● Hyperendemic setting ● Clinical grading: simplified WHO system ● Chlamydia test: Amplicor PCR, Roche
Gambia, 2006, [40]	Cross-sectional population-based study of school children aged 4–15 years from nine villages.	4–15 years: 331	4–15 years: ● TF 18.2% (60/331) ● TI 1.5% (5/331)	4–15 years: 21.8% (72/331)	4–15 years: 41.7% (25/60)	4–15 years: 15.3% (38/249)	● Mesoendemic setting ● Clinical grading: simplified WHO system ● Chlamydia test: in-house RT-PCR assay for chlamydial 16S rRNA
Tanzania, 2006^a^, [41]	Cross-sectional population-based study of children aged 1–9 years from two hyperendemic villages.	1–9 years: 464	1–9 years: ● TF 44.0% (204/464)	1–9 years: 24.8% (115/464)	1–9 years: 36.3% (74/204)	1–9 years: 13.6% (41/301)	● Hyperendemic setting ● Clinical grading: simplified WHO system ● Chlamydia test: Amplicor PCR, Roche
Tanzania, 2006^b^, [41]	Cross-sectional population-based study of children aged 1–9 years from one hypoendemic village.	1–9 years: 200	1–9 years: ● TF 12.5% (25/200)	1–9 years: 6.5% (13/200)	1–9 years: 32.0% (8/25)	1–9 years: 2.9% (5/175)	● Hypoendemic setting ● Clinical grading: simplified WHO system ● Chlamydia test: Amplicor PCR, Roche
Niger, 2007, [42]	Cross-sectional study of randomly selected children from 12 villages.	1–5 years: 651	1–5years: ● TF/TI 43.0% (280*/651) ● TI 16% (104*/651)	1–5years: 21.0% (137*/651)	1–5years: 37.3% (103*/267)	1–5years: 9.0% (33*/365)	● Hyperendemic setting ● Clinical grading: simplified WHO system ● Chlamydia test: Amplicor PCR, Roche
Ethiopia, 2007, [43]	Cross-sectional population-based study of children aged 0–10 years from two hyperendemic villages.	0–10 years: 56	0–10 years: ● TF/TI 78.6% (44/56) ● TI 46.4% (26/56)	0–10 years: 39.3% (22/56)	0–10 years: 43.1% (19/44)	0–10 years: 25.0% (3/12)	● Hyperendemic setting ● Clinical grading: simplified WHO system ● Chlamydia test: Amplicor PCR, Roche
Nepal, 2008, [44]	Cross-sectional study of 9 randomly selected households from one village	All ages: 127	All ages: ● TF 26.8% (34/127) ● TI 38.6% (49/127)	All ages: 38.6% (49/127)	All ages: 44.1% (15/34)	All ages: 36.6% (34/93)	● Hyperendemic setting ● Clinical grading: simplified WHO system ● Chlamydia test: Amplicor PCR, Roche
Gambia, 2009, [45, 46]	Cross-sectional survey using a two-stage cluster random sampling strategy with probability of selection proportional to size, in 19 enumeration areas in Lower River Region, The Gambia.	1–9 years: 876	1–9 years: ● TF 12.3% (108/876) ● TI 0.1% (1/876)	1–9 years: 0.3% (3/876)	1–9 years: 0.9% (1/108)	1–9 years: 0.3% (2/768)	● Mesoendemic setting ● Clinical grading: simplified WHO system ● Chlamydia test: Amplicor PCR, Roche
Ethiopia, 2009, [47]	Cross-sectional population-based sample of 8 randomly selected children (1–5 years) per village from eight villages.	1–5 years: 120	1–5 years: ● TF/TI 60.8% (73/120) ● TI 44.2% (53/120)	1–5 years: 40.8% (49/120)	Data not available	Data not available	● Hyperendemic setting ● Clinical grading: simplified WHO system ● Chlamydia test: Amplicor PCR, Roche
Ethiopia, 2010, [18, 48]	Cross-sectional population-based sample of children (1–5 years) living in 24 villages.	1–5 years: 1200	1–5 years: ● TF/TI 86.0% (mean village prevalence)	1–5 years: 52.9% (mean village prevalence)	Data not available	Data not available	● Hyperendemic setting ● Clinical grading: simplified WHO system ● Chlamydia test: Amplicor PCR, Roche ● NB: The data are reported a mean of the prevalence across all 24 villages.
Gambia, 2010, [25, 49]	Cross-sectional population-based survey of ~100 randomly selected children aged 0–5 years from 48 enumeration areas.	0–5 years: 5033	0–5 years: ● TF 6.3% (316/5033) ● TI 0.6% (28/5033)	0–5 years: 0.8% (39/5033)	0–5 years: 0.9% (3/316)	0–5 years: 0.7% (36/4717)	● Hypoendemic setting ● Clinical grading: simplified WHO system ● Chlamydia test: Amplicor PCR, Roche.
Tanzania, 2010, [49–51]	Cross-sectional population-based survey of ~100 randomly selected children aged 0–5 years from 32 enumeration areas. The villages were purposely selected based on having a preliminary survey prevalence of >20%.	0–5 years: 3122	0–5 years: ● TF 30.8% (963/3122) ● cTI 7.8% (244/3122)	0–5 years: 21.9% (684/3122)	0–5 years: 48.9% (471/963)	0–5 years: 9.8% (213/2159)	● Hyperendemic setting ● Clinical grading: simplified WHO system ● Chlamydia test: Amplicor PCR, Roche.
Niger, 2010, [52]	Cross-sectional study of randomly selected children from 12 villages	1–5 years: 557	1–5 years: ● TF/TI 41.6% (232/557)	1–5 years: 20.1% (112/557)	Data not available	Data not available	● Hyperendemic setting ● Clinical grading: simplified WHO system ● Chlamydia test: Amplicor PCR, Roche
Australia, 2011, [14]	Population-based cross-sectional study in five Aboriginal communities.	All ages: 1282	All ages: ● TF 8.4% (108/1282)	All ages: 3.6% (46/1282)	All ages: 17.6% (19/108)	All ages: 2.3% (27/1174)	● Hypoendemic setting ● Clinical grading: variation of the detailed WHO-FPC system [14] ● Chlamydia test: Amplicor PCR, Roche
Tanzania, 2011, [26, 53]	Cross-sectional population-based survey in three villages	0–9 years: 473	0–9 years: ● TF 13.7% (65/473) ● TI 1.3% (6/473)	0–9 years: 5.3% (25/473)	0–9 years: 6.1% (4/65)	0–9 years: 5.1% (21/408)	● Mesoendemic setting ● Clinical grading: detailed WHO-FPC system ● Chlamydia test: Amplicor PCR, Roche.
Tanzania, 2011, [54, 55]	Cross-sectional baseline population-based survey in 4 villages.	0–8 years: 1991	0–8 years: ● TF 27.8% (553/1991)	0–8 years: 23.7% (463/1956)	Data not available	Data not available	● Hyperendemic setting ● Clinical grading: simplified WHO system ● Chlamydia test: Amplicor PCR, Roche
Tanzania, 2011, [56]	Cross-sectional baseline, population-based survey of four communities	0–9 years: 2118	0–9 years: ● TF/TI 27.7%: (586/2118)	0–9 years: 23.6% (499/2118)	0–9 years: 33.1% (194*/586)	0–9 years: 13.6% (209/1532)	● Hyperendemic setting ● Clinical grading: simplified WHO system ● Chlamydia test: Amplicor PCR, Roche
Ethiopia, 2011, [57]	Cross-sectional population-based survey in 23 communities. Both arms of a cluster RCT at baseline.	0–9 years: 1168	0–9 years: ● TF/TI 66.5% (770/1158)	0–9 years: 44.7% (516/1168)	Data not available	Data not available	● Hyperendemic setting ● Clinical grading: simplified WHO system ● Chlamydia test: Amplicor PCR, Roche
Ethiopia, 2012, [19, 58, 59]	Cross-sectional baseline, population-based survey of 0–9 year olds in 12 communities	0–9 years: 583	0–9 years: ● TF/TI 68.3% (393/575)	0–9 years: 42.4% (248/584)	Data not available	Data not available	● Hyperendemic setting ● Clinical grading: simplified WHO system ● Chlamydia test: Amplicor PCR, Roche
Cameroon, 2012, [24]	Cross-sectional study, with a random selection of children from 30 villages, with probability proportional to size. Only children with signs of Active Trachoma were tested for infection by PCR.	1–9 years: 2397	0–9 years: ● TF/TI 26.2% (628/2397) ● TI 5.2% (124/2397)	Data not available	0–9 years: 35.0% (220*/628)	Data not available	● Hyperendemic setting ● Clinical grading: simplified WHO system ● Chlamydia test: in-house PCR assay for chlamydial rRNA gene.
Niger, 2012, [60, 61]	Cross-sectional study for randomly selected children from 48 communities.	0–5 years: 4484	0-5years: ● TF 26.0% (1166*/4484)	0-5years: 20.7% (928*/4484)	Data not available	Data not available	● Hyperendemic setting ● Clinical grading: simplified WHO system ● Chlamydia test: Amplicor PCR, Roche
Guinea Bissau, 2013, [62, 63]	Cross-sectional population-based survey across multiple communities	● All ages: 1508 ● 1–9 years: 618	All ages: ● TF/TI 11.1% (167/1508) ● TI 2.0% (29/1508)1–9 years: ● TF/TI 22.0% (136/618) ● TI 3.2% (21/618)	● All ages: 17.9% (269/1507) ● 1–9 years: 25.4% (157/618)	● All ages: 63.3% (100/158) ● 1–9 years: 86/129 (66.7%)	● All ages: 12.1% (164/1351) ● 1–9 years: 83/537 (15.5%)	● Mesoendemic setting ● Clinical grading: simplified WHO system ● Chlamydia test: Amplicor PCR, Roche
Tanzania, 2013, [64]	Cross-sectional population-based survey of children from one village.	0–9 years: 27	0–9 years: ● TF 44.9% (57/127) ● TI 16.5% (21/127)	0–9 years: 27.6% (35/127)	0–9 years: 29/57 (50.9%)	0–9 years: 6/70 (8.6%)	● Hyperendemic setting ● Clinical grading: simplified WHO system ● Chlamydia test: Amplicor PCR, Roche.
Tanzania, 2014, [65]	Cross-sectional population-based survey of all 1–6 year olds in a single village	1–6 years: 208	1–6 years: ● TF/TI 47.0% (98/208)	1–6 years: 25.0% (52/208)	Data not available	Data not available	● .Hyperendemic setting ● Clinical grading: simplified WHO system ● Chlamydia test: Amplicor PCR, Roche.

* Estimated value inferred from available data in publication.

**Table 2:** Active Trachoma and *Chlamydia trachomatis* (Ct) infection after the introduction of mass antibiotic treatment.

Country, Year	Study design	Time Point post 1^st^ MDA	Participants	Active Trachoma %	Ct %	Ct+/TF+	Ct+/TF-	Comments
Tanzania, 1993, [20, 66]	Cross-section survey of randomly selected children one month after the completion of a one month tetracycline treatment course, given as MDA to the entire community.	1 month	1–7 years: 227	1–7 years: ● TF/TI 41.9% (95/227) ● TI 2.6% (6/227)	1–7 years: 23.8% (54/227)	Data not available	Data not available	● Hyperendemic setting ● Clinical grading: simplified WHO system ● Chlamydia test: in-house PCR-EIA for *OMP1*.
Egypt, 2003, [10, 31]	Cross-section population-based survey 14 months after azithromycin MDA.	14 months	1–10 years: 354	1–10 years: ● TF/TI 26.0% (92/354)	1–10 years: 5.1% (18/354)	1–10 years: 9.8% (9/92)	1–10 years: 3.4% (9/262)	● Hyperendemic setting ● Clinical grading: detailed WHO-FPC system ● Chlamydia test: LCR, Abbott Laboratories. ● Only the azithromycin arm is included.
Gambia, 1999, [31]	Cross-section population-based survey 12 months after azithromycin MDA.	12 months	All ages: ● 675 (exam) ● 540 (tested)	All ages: ● TF/TI 8.6% (58*/675) ● TI 2.2% (15*/675)	All ages: 8.3% (45/540)	Data not available	Data not available	● Hyperendemic setting, pre treatment ● Clinical grading: detailed WHO-FPC system ● Chlamydia test: LCR, Abbott Laboratories. ● Only the azithromycin arm is included.
Tanzania, 1999, [31]	Cross-section population-based survey 12 months after azithromycin MDA.	12 months	All ages: ● 1308 (exam) ● 1162 (tested)	All ages: ● TF/TI 24.6% (322*/1308) ● TI 6.3% (83*/1308)	All ages: 7.0% (82/1162)	Data not available	Data not available	● Hyperendemic setting ● Clinical grading: detailed WHO-FPC system ● Chlamydia test: LCR, Abbott Laboratories. ● Only the azithromycin arm is included.
Nepal, 2001, [23]	Cross-section survey of randomly selected normal children and a purposive sample of children with Active Trachoma, 6 months after azithromycin MDA.	6 months	1–7 years: ● 5262 (exam) ● 394 (tested)	1–7 years: ● TF/TI 16% (841*/5262)	Data not available	1–7 years: 11.8% (31/263)	1–7 years: 5.1% (6/118)	● Mesoendemic setting ● Clinical grading: simplified WHO system ● Chlamydia test: LCR, Abbott Laboratories.
Tanzania, 2004, [34, 35]	Cross-sectional survey of all residents of a sub-village, 24 months after MDA	24 months	All ages: 842	All ages: ● TF/TI 5.8% (49/842)	All ages: 0.1% (1/842)	All ages: 2.0% (1/49)	All ages: 0.0% (0/793)	● Hyperendemic setting, pre-treatment ● Clinical grading: simplified WHO system ● Chlamydia test: Amplicor PCR, Roche
Gambia, 2005, [7, 27, 32, 33]	Cross-sectional survey of all residents of 14 small villages, 12 months after MDA	12 months	● All ages: 1210 ● 1–9 years: 440	All ages: ● TF/TI 3.9% (47/1210)1–9 years: ● TF 6.8% 30/440) ● TI 0.9% (4/440)	All ages: 2.3% (28/1210)1–9 years: 5.4% (24/440)	All ages: 29.8% (14/47)1–9 years: 36.7% (11/30)	All ages: 1.2% (14/1163)1–9 years: 3.2% (13/410)	● Mesoendemic setting, pre-treatment ● Clinical grading: detailed WHO-FPC system ● Chlamydia test: Amplicor PCR, Roche.
Tanzania, 2005, [27, 36–39]	Cross-sectional survey of residents of a village, 12 months after MDA	12 months	0–7 years: 287	0–7 years: ● TF/TI 46.8% (134*/287)	0–7 years: 12.8% (37*/287)	Data not available	Data not available	● Hyperendemic ● Clinical grading: simplified WHO system ● Chlamydia test: Amplicor PCR, Roche
Tanzania, 2007, [39]	Cross-sectional of population-based survey 5 years after baseline MDA and 3.5 years after a second MDA.	5 years	0–10 years: 464	0–10 years: ● TF 30.2% (140/464) ● TI 10.6% (49/464)	0–10 years: 25.9% (120/464)	Data not available	Data not available	● Hyperendemic setting ● Clinical grading: simplified WHO system ● Chlamydia test: Amplicor PCR, Roche
Ethiopia, 2008, [67]	Cross-sectional population based sample of children from 32 communities. These had received between 1 and 3 rounds of MDA, with the most recent does less than 6 months in about half the communities.	Variable	3–9 years: 1459	3–9 years: ● TF 23.6% (345/1459)	3–9 years: 3.0% (44/1459)	3–9 years: 6.1% (21/345)	3–9 years: 6.1% (23/1114)	● Hyperendemic setting ● Heterogeneous treatment history ● Clinical grading: simplified WHO system ● Chlamydia test: Amplicor PCR, Roche
Ethiopia, 2009, [47]	Cross-sectional population-based sample of 8 randomly selected children (1–5 years) per village from eight villages. The villages had received MDA biannually for 2 years, with the last dose 18 months prior to the survey.	42 months	0–5 years: 120	0–5 years: ● TF/TI 47.5%: (57/120) ● TI 30.0% (36/120)	0–5 years: 15% (18/120)	Data not available	Data not available	● Hyperendemic setting ● Clinical grading: simplified WHO system ● Chlamydia test: Amplicor PCR, Roche
Ethiopia, 2010, [18, 48]	Cross-sectional population-based sample of children (1–5 years) living in 24 villages. Communities had received 4 biannual MDA, the most recent 6 months before survey	24 months	1–5 years: 1234	1–5 years: ● TF/TI 39.2% (mean village prevalence)	1–5 years: 2.0% (mean village prevalence)	Data not available	Data not available	● Hyperendemic setting ● Clinical grading: simplified WHO system ● Chlamydia test: Amplicor PCR, Roche ● NB: The data are reported a mean of the prevalence across all 24 villages.
Tanzania, 2011, [15]	Cross-sectional population-based sample of children from 71 communities. All communities had received 3 to 7 rounds of MDA.	Variable	0–5 years: 7817	0–5 years: ● TF 10.0% (784/7817) ● TI 3.2% (252/7817)	0–5 years: 5.5%% (429/7817)	0–5 years: 23.5% (184/784)	0–5 years: 3.5% (245/7033)	● Mesoendemic setting ● Clinical grading: simplified WHO system ● Chlamydia test: Amplicor PCR, Roche Heterogeneous MDA history. Wide range of community level prevalence of infection and disease. Summary data here pools these communities
Ethiopia, 2011, [57]	Cross-sectional population-based survey in 24 communities. Both arms of a cluster RCT of latrine provision received a single round of MDA at baseline.	24 months	0–9 years: 1211	0–9 years: ● TF/TI 47.0% (567/1207)	0–9 years: 14.6% (177/1211)	Data not available	Data not available	● Hyperendemic setting ● Clinical grading: simplified WHO system ● Chlamydia test: Amplicor PCR, Roche ● Data from both arms combined.
Ethiopia, 2012, [19, 58, 59, 68, 69]	Cross-sectional survey of 50 children per village under 10 years in 12 villages. Three annual rounds of MDA were given. The final survey was months after the last MDA.	36 months	0–9 years: 577	0–9 years: ● TF 43.5% (251/577)	0–9 years: 4.3% (25/577)	Data not available	Data not available	● Hyperendemic setting ● Clinical grading: simplified WHO system ● Chlamydia test: Amplicor PCR, Roche
Gambia, 2013, [25, 70]	Cross-sectional population-based sample of children from 48 communities enrolled in an cluster RCT from four districts. 24 communities had 3 annual MDA and 24 communities had a single round of MDA at baseline. Survey was 3 years after the first round of MDA.	36 months	0–5 years: ● District 1: 1128 ● District 2: 1199 ● District 3: 1243 ● District 4: 1246	0–5 years: ● TF 0.2% (2/1128) ● TF 0.3% (3/1199) ● TF 3.8% (47/1243) ● TF 6.4% (80/1246)	0–5 years: ● 0.5% (6/1128) ● 0.1% (1/1198) ● 1.1% (13/1241) ● 0.3% (4/1235)	0–5 years: ● 0.0% (0/2) ● 0.0% (0/3) ● 6.4% (3/47) ● 0.0% (0/80)	0–5 years: ● 0.5% (6/1126) ● 0.1% (1/1196) ● 0.8% (10/1196) ● 0.3% (4/1166)	● Hypoendemic setting ● Clinical grading: simplified WHO system ● Chlamydia test: Amplicor PCR, Roche. ● Data from both trial arms were combined, and presented separately for each district.
Tanzania, 2014, [51, 71]	Cross-sectional survey of all children under 10 years in four villages. Four annual rounds of MDA were given. The final survey was 6 months after the last MDA.	42 months	0–9 years: 2234	0–9 years: ● TF/TI 7.9% (176/2234)	0–9 years: 5.1% (114/2234)	Data not available	Data not available	● Hyperendemic setting, pre-treatment ● Clinical grading: simplified WHO system ● Chlamydia test: Amplicor PCR, Roche
Tanzania, 2014, [71, 72]	Cross-sectional population-based sample of children aged 0–5 years in 32 villages, 100 children per village. Survey was done 12 months after the third MDA.	36 months	0–5 years: 3136	0–5 years: ● TF 7.6% (237/3136)	0–5 years: 4.5% (142*/3136)	0–5 years: 29.5% (70/237)	0–5 years: 2.5% (72*/2899)	● Hyperendemic setting, pre-treatment ● Clinical grading: simplified WHO system ● Chlamydia test: Amplicor PCR, Roche
Tanzania,: 2015,[73]	Cross-sectional population-based of residents of a village, which had received 2 rounds of MDA 12 and 10 years previously.	12 years	● All ages: 571 ● 1–9 years: 200	All ages: ● TF 2.5% (14/571)1–9 years: ● TF 6.5% (13/200) ● TI 1.5% (3/200)	All ages: 0% (0/571)1–9 years: 0% (0/200)	Data not available	Data not available	● Hyperendemic setting, pre-treatment ● Clinical grading: simplified WHO system ● Chlamydia test: Amplicor PCR, Roche
Nepal, 2016,[74]	Cross-section survey of 1–9 year olds in 24 randomly selected communities. Four rounds of MDA. The survey was conducted 5 years after the first round.	5 years	1–9 years: 1124	1–9 years: ● TF/TI 0.3% (3/1124)	1–9 years: 0% (0/1124)	Data not available	Data not available	● Hypoendemic setting ● Clinical grading: simplified WHO system ● Chlamydia test: Amplicor PCR, Roche
Tanzania, 2016, [75]	Random sample of children aged 1–9 years, from 30 hamlets. 50 children were sampled per hamlet. Communities had received variable rounds of MDA, between 4 and seven years previously.	7 years	1–9 years: 1474	1–9 years: ● TF 0.4% (6*/1474)	1–9 years: 1.1% (16/1474)	Data not available	Data not available	● Hypoendemic setting ● Clinical grading: simplified WHO system ● Chlamydia test: Aptima Combo2, Hologic

* Estimated value inferred from available data in publication.

^a^ Additional data sub-divided by district were provided by the authors of this study.

### Pre-Treatment

Of the 36 pre-treatment study populations, 24 were hyperendemic, 8 mesoendemic and 4 hypoendemic (Table 1). The studies were conducted in populations from Tanzania (12), The Gambia (7), Ethiopia (6), Nepal (4), Niger (3), Cameroon (1), Guinea Bissau (1), Egypt (1) and Australia (1). The Simplified WHO Trachoma Grading System was used in 28 studies and 8 used the Detailed WHO FPC System. The majority of studies (25) used the commercially produced *Amplicor CT/NG* (Roche) PCR assay, five used ligase chain reaction (LCR, Abbott Laboratories), and six used in-house PCR assays. Four of the 36 studies listed in Table 1 did not report the community prevalence of *C*. *trachomatis* infection, however they report infection on a selected subset of individuals [11, 22–24]. These four studies were therefore retained in Table 1 as they contribute useful information, however they were excluded from the meta-analysis. Of the remaining 32 studies included in the meta-analysis, 29 studies reported data for childhood age groups (variable age ranges), seven of which also reported all age data, and three studies reported only all age data.

**Fig 2:**
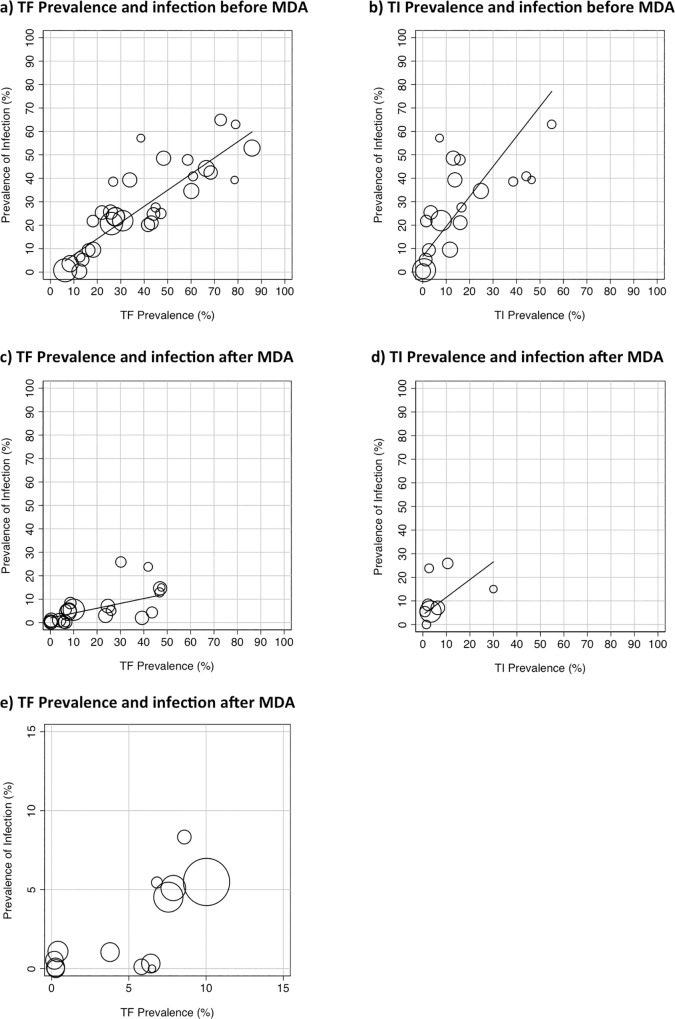
The relationship between the prevalence of disease signs and infection before and after the introduction of MDA. (a) Community prevalence of TF (or TF/TI) vs. the community prevalence of *C*. *trachomatis* infection before the introduction of MDA. (b) Community prevalence of TI vs. the community prevalence of *C*. *trachomatis* infection before the introduction of MDA. (c) Community prevalence of TF (or TF/TI) vs. the community prevalence of *C*. *trachomatis* infection after the introduction of MDA. (d) Community prevalence of TI vs. the community prevalence of *C*. *trachomatis* infection after the introduction of MDA. (e) Community prevalence of TF (or TF/TI) vs. the community prevalence of *C*. *trachomatis* infection after the introduction of MDA, showing only the communities with less than 15% TF. Data from population-based studies, summarised in Tables 1 and 2. The size of the circles reflects the sample size. Line fitted by linear regression, weighted by the size of the studies.

Prior to the introduction of MDA there was evidence of a strong positive correlation between the community-level prevalence of Active Trachoma and the community-level prevalence of detected *C*. *trachomatis*, using data from all 32 studies (r = 0.92, 95%CI 0.83 to 0.96, p<0.0001), Fig 2A. The correlation was similar when the analysis was limited to children only data (29 studies; r = 0.91, 95%CI 0.82 to 0.96, p<0.0001). The correlation was much weaker for all age data (10 studies; r = 0.51, 95%CI -0.18 to 0.86 p = 0.13). Overall, the community prevalence of TF is typically greater than the underlying community prevalence of detected *C*. *trachomatis*, with TF having a higher prevalence than *C*. *trachomatis* in 25 out of the 32 studies. The mean difference in prevalence was 10.4% (95% CI 5.9%-15.0%, p = 0.001).

There were 19 studies that reported the prevalence of TI separately from TF. There was evidence of a moderate positive correlation between the community-level prevalence of TI and the community-level prevalence of detected *C*. *trachomatis* (r = 0.75, 95%CI 0.45 to 0.90 p = 0.0002), Fig 2B. Although there are only a limited number of studies, in all communities where the TI prevalence was >20% the prevalence of *C*. *trachomatis* was at least 30%. In contrast, where the TI prevalence was below 20% there was a considerable degree of variation in the underlying community prevalence of infection.

**Fig 3:**
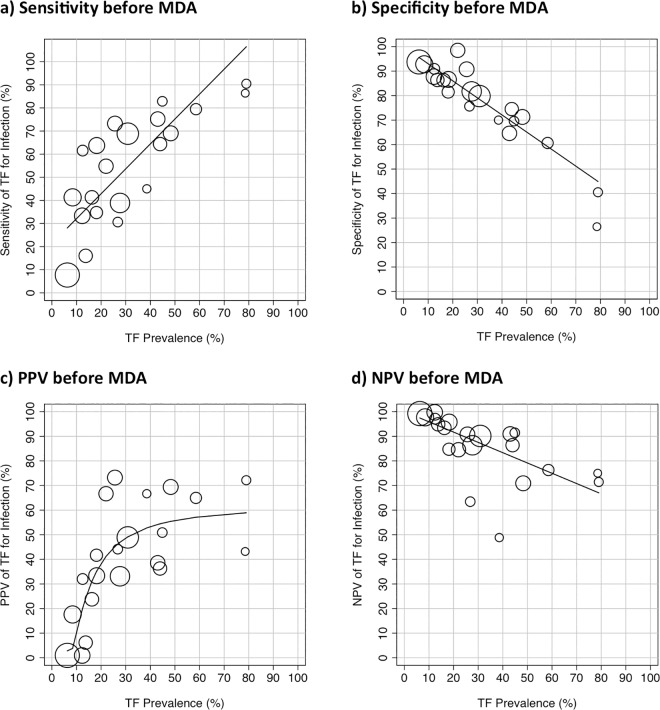
The relationship between the individual level presence of Active Trachoma (TF or TF/TI) and the detection of *C*. *trachomatis* infection by community TF prevalence before the introduction of MDA. (a) Sensitivity of TF for infection. (b) Specificity of TF for infection. (c) Positive Predictive Value (PPV) of TF for *C*. *trachomatis* infection. (d) Negative Predictive Value (NPV) of TF for *C*. *trachomatis* infection. Data from population-based studies, summarised in Table 1. The size of the circles reflects the sample size. Line fitted by linear regression, weighted by the size of the studies, except for the PPV which was fitted by polynomial.

**Fig 4:**
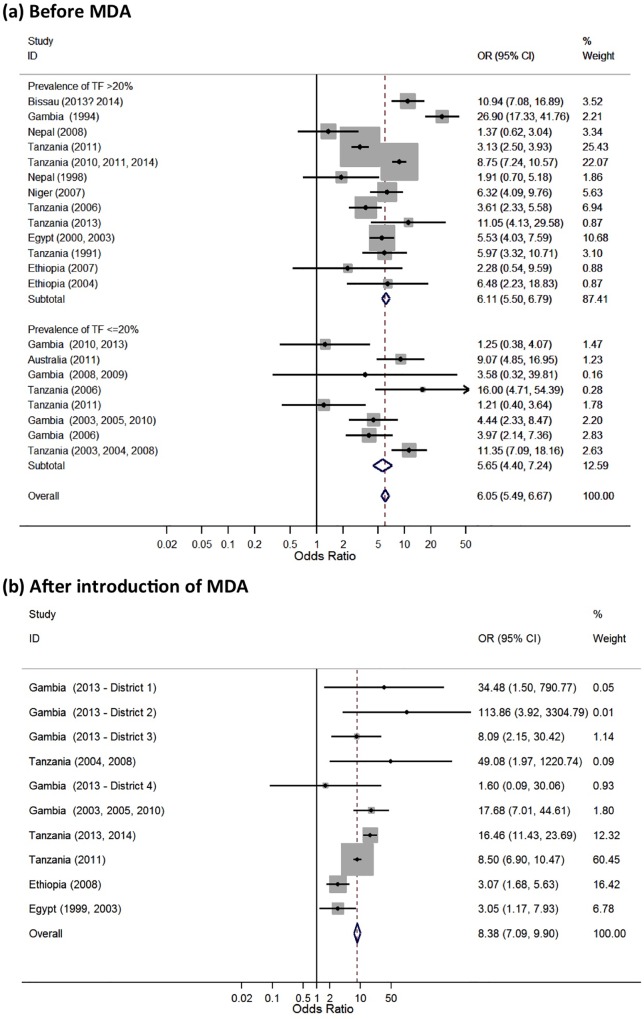
A Forest plot showing the relationship between Active Trachoma (TF or TF/TI) and the detection of *C*. *trachomatis* infection at the individual level (a) before and (b) after the introduction of MDA, grouped by community TF prevalence level. Studies are ordered by increasing prevalence of TF, the size of the grey boxes represent the how much weight each study contributes to the overall estimate, the blue diamonds represent the subtotal and overall pooled odds ratio estimates. The odds ratios are for ocular *C*. *trachomatis* infection as the outcome and TF as the explanatory variable.

**Fig 5:**
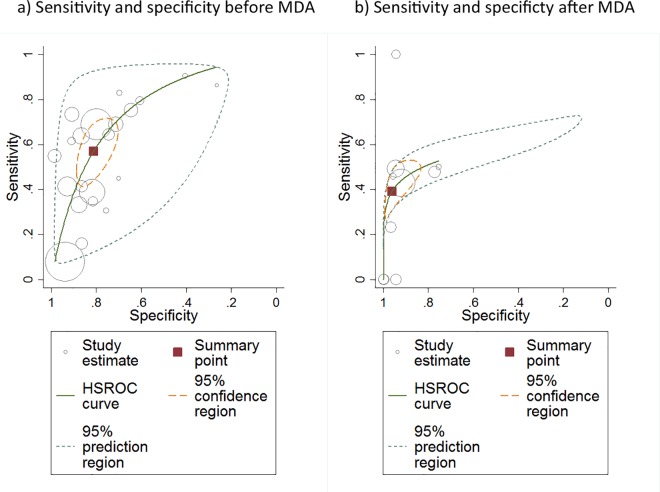
Sensitivity versus specificity of each study, using TF to diagnose *C*. *trachomatis* infection at the individual level (a) before and (b) after the introduction of MDA. Each circle represents the estimate for a single study, with the size of the circle representing the size of the study. The red square is the estimated pooled sensitivity and specificity for all studies (Pre- or Post-MDA). The orange dashed line represents the 95% CI (or confidence region in 2 dimensions) for the sensitivity and specificity. The grey curve (hierarchical summary receiver operating characteristic (HSROC) curve) represents the estimated relationship between sensitivity and specificity in these studies, with the grey dashed line indicating the region in which we would expect 95% of studies to fall.

There was sufficient data at the individual level presented to estimate the sensitivity, specificity, PPV and NPV for 21 studies. The sensitivity of TF for identifying the presence of *C*. *trachomatis* infection varied substantially with the prevalence of TF in the community, Fig 3A. There was evidence of a strong positive correlation (r = 0.82, 95%CI 0.59 to 0.92, p<0.0001). In contrast, there was a strong negative correlation between the specificity of TF for infection and the community prevalence of TF (r = -0.92, 95%CI -0.97 to -0.80, p<0.0001), Fig 3B. The Forest plot of the pre-treatment relationship between disease and detection of infection at the individual level showed an overall strong association (OR 6.05, 95% CI 5.49 to 6.67, p<0.0001), although there was marked heterogeneity (I^2^ = 87.1%, p<0.001), Fig 4A. A plot of sensitivity against specificity of the pre-treatment studies using TF as a test for *C*. *trachomatis* infection at the individual level showed that the overall estimated sensitivity was moderate (57.1% (45.4–68.1%) and the overall specificity was good (81.1% (73.4–87.0%), Fig 5A.

The relationship between the community-level prevalence of Active Trachoma and the proportion of people with TF who were infected with *C*. *trachomatis* (Positive Predictive Value, PPV) showed a positive correlation (r = 0.80, 95%CI 0.56 to 0.91, p<0.0001), Fig 3C. The pattern of this distribution suggests that when the community-level prevalence of TF is below 20% the PPV of TF for the presence of *C*. *trachomatis* infection drops substantially. Above a community-level prevalence of TF of 20% the PPV is typically 40–70%, across a wide range of TF prevalence.

The relationship between the community-level prevalence of Active Trachoma and the proportion of people without TF who were not infected with *C*. *trachomatis* (Negative Predictive Value, NPV) showed a strong negative correlation (r = -0.81, 95%CI -0.92 to -0.57, p<0.0001), Fig 3D. When the community-level TF prevalence was greater than 20%, the NPV was more variable.

### Post-Treatment

Of the 21 studies from populations following the introduction of MDA, prior to the first treatment, 15 were hyperendemic, 3 mesoendemic and 3 hypoendemic (Table 2). The studies were conducted in populations from Tanzania (10), Ethiopia (5), The Gambia (3), Nepal (2), and Egypt (1). The Simplified WHO Trachoma Grading System was used in 17 studies and four used the Detailed WHO FPC System. The majority of studies (15) used the Amplicor CT/NG (Roche) PCR assay, four used LCR (Abbott), one used an in-house PCR and one used the Aptima Combo2 (Hologic) TMA assay. There was one study included in Table 2 that did not report the community prevalence of *C*. *trachomatis* infection, and was therefore not included in the meta-analysis [23]. One of the Gambian studies involved surveys in four separate districts; we have included these as four separate sets of data in the analysis [25]. This resulted in 23 discrete studies included in the meta-analysis: for 20 studies data was available on children only (variable age ranges), two of which also reported all age data, and three other studies reported only all age data.

Following the introduction of MDA there was evidence of a moderate positive correlation between the community-level prevalence of Active Trachoma and the community-level prevalence of detected *C*. *trachomatis*, using data from all 23 studies (r = 0.60, 95%CI 0.25 to 0.81, p = 0.003), Fig 2C. The relationship was similar when the analysis was limited to the 20 studies of children only (r = 0.60, 95%CI 0.21 to 0.82, p = 0.005). However, for the five studies reporting results for all ages there was no significant correlation (r = 0.72, 95%CI -0.18 to 0.86 p = 0.18). The overall impression is that the relationship between disease and infection is more uncertain post-MDA, such that the community-level prevalence of TF can substantially overestimate the underlying community-level prevalence of *C*. *trachomatis*; the community prevalence of TF can remain high (>20%) even when the prevalence of infection has declined (<10%), Fig 2C and 2E.

We found evidence that the relationship between the prevalence of TF and the prevalence of *C*. *trachomatis* infection differs between pre and post-MDA. Assuming a linear relationship between *C*. *trachomatis* and TF, the prevalence of *C*. *trachomatis* was associated with an expected 6.5% increase for every 10% increase in TF in pre-MDA studies, compared with an increase of 2.8% in post-MDA (test for interaction p = 0.004).This demonstrates that as the prevalence of TF increases, the expected increase in *C*. *trachomatis* is higher in pre-MDA communities than in post-MDA communities.

There were only eight studies that reported the prevalence of TI separately from TF after the introduction of MDA. There was no evidence of a correlation between the community-level prevalence of TI and the community-level prevalence of detected *C*. *trachomatis* (r = 0.50, 95%CI -0.31 to 0.89 p = 0.20), Fig 2D. However, it is noteworthy that the prevalence of TI was low (<10%) in all but two communities.

**Fig 6:**
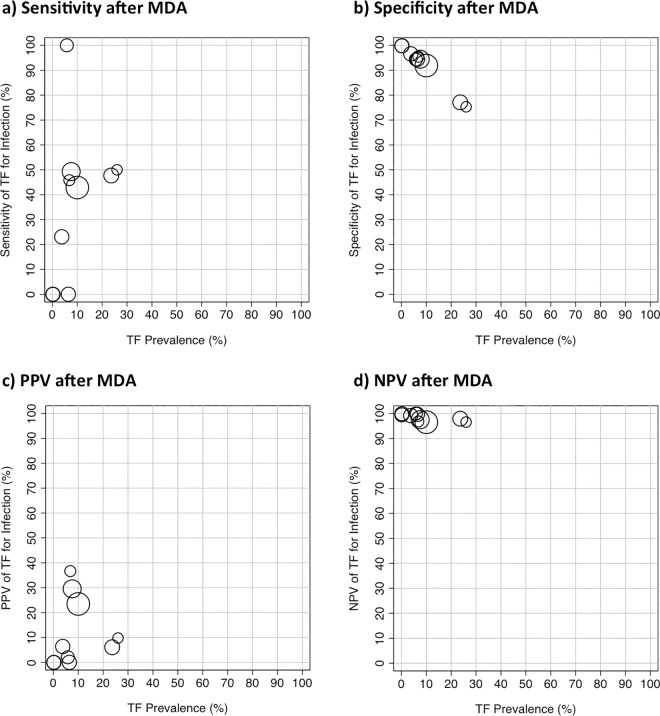
The relationship between the individual level presence of Active Trachoma (TF or TF/TI) and the detection of *C*. *trachomatis* infection by community TF prevalence after the introduction of MDA. (a) Sensitivity of TF for infection. (b) Specificity of TF for infection. (c) Positive Predictive Value (PPV) of TF for *C*. *trachomatis* infection. (d) Negative Predictive Value (NPV) of TF for *C*. *trachomatis* infection. Data from 6 population-based studies, summarised in Table 2. The size of the circles reflects the sample size.

There was sufficient data presented to estimate the sensitivity, specificity, PPV and NPV for only 10 studies at the individual level after introduction of MDA. Therefore, only limited inference can be drawn. The sensitivity of TF for identifying the presence of *C*. *trachomatis* infection after the introduction of MDA varied widely, across a range of community-level TF prevalence levels, Fig 6A. In contrast, there was evidence of a strong negative correlation between the specificity of TF for infection and the community prevalence of TF (r = -0.99, 95%CI -0.99 to -0.96, p<0.0001), Fig 6B. The Forest plot of the post-treatment relationship between disease and detection of infection at the individual level showed an overall strong association (OR 8.38, 95% CI 7.09 to 9.90, p<0.0001), although there was marked heterogeneity (I^2^ = 75%, p<0.001), Fig 4B. Although this overall OR is slightly higher post-MDA compared to pre-MDA, it should be noted that this result is calculated from individual-level data, whereas the correlations between TF and *C*. *trachomatis* infection prevalence (Fig 2) are calculated from population-level data. Compared to the pre-MDA situation (Fig 5A), after the introduction of MDA the individual level sensitivity was weaker (39.2% (30.9%, 48.2%) while specificity was stronger (96.1% (89.6%, 98.6%), Fig 5B.

The community-level prevalence of Active Trachoma and the proportion of people with TF who were infected with *C*. *trachomatis* (PPV) did not appear to be correlated (r = 0.16, 95%CI -0.52 to 0.72, p = 0.65), Fig 6C. Finally, the proportion of people without TF who were not infected with *C*. *trachomatis* (NPV) was high (>90%), across the limited range of community-level prevalence of Active Trachoma in these studies, Fig 6D.

## Discussion

It has long been observed that the relationship between the signs of Active Trachoma and the detection of *C*. *trachomatis* infection at the individual level is not highly concordant [12]. Surveys, including those in this review, consistently find that within endemic populations there are many individuals with signs of disease who do not have detectable infection and conversely there are people who do not meet the diagnostic criteria for Active Trachoma (TF or TI) who do have detectable infection. Therefore, at the individual level, signs of infection cannot be depended upon to determine which members of an endemic community have ocular *C*. *trachomatis* infection.

In a trachoma endemic population, the main reason for this mismatch between active disease and infection is probably the different time courses of the typical infection and disease episodes, outlined in the introduction and illustrated in Fig 1. In addition, it is also possible that other factors contribute to this mismatch. Some individuals who have detectable infection but do not meet the diagnostic criteria for TF may have a mild trachomatous follicular conjunctivitis. Others may have previously acquired immunity and are able clear the infection without developing any detectable inflammatory signs. Alternatively, a positive NAAT test for *C*. *trachomatis* in a clinically normal individual could arise through cross-contamination during sample collection or processing. Clinical signs similar to those of Active Trachoma can also arise for other reasons, such as viral or bacterial infections, vernal conjunctivitis and hypersensitivity reactions [26].

It is clear that clinical signs are not a reliable indicator for *C*. *trachomatis* at the individual level. There is no point-of-care diagnostic test available for programmes to use to determine which individual members of a community are infected, and who would therefore benefit from targeted antibiotic treatment. Moreover, a strategy of testing everybody is not considered a practical option. Therefore, the WHO guidance and the standard practice is to conduct community-wide antibiotic distribution of the entire population of endemic districts.

Decisions around the initiation and cessation of trachoma control measures are based on the prevalence of TF in children aged 1–9 years, determined through district level surveys, such as those conducted by the Global Trachoma Mapping Project. District-wide antibiotic treatment programmes and F&E measures to suppress transmission are initiated where the initial prevalence of TF is ≥10%. Below 10% TF the advice is to conduct sub-district level surveys. If a sub-district has ≥10% TF MDA and F&E are implemented. For sub-districts between 5% and 9.9% TF the guidance is to consider targeted MDA and F&E measures. For sub-districts <5% no MDA is needed and implementation of F&E can be considered.

The dependence on the clinical signs of disease to guide programmatic decisions raises the important question of how accurate these clinical signs are as a proxy measure for *C*. *trachomatis* infection at the population level. This question is particularly relevant after the introduction of MDA, when the association between clinical signs and infection prevalence at the population level is weaker, and as we try to determine when elimination targets have been reached.

In this systematic review, we found that prior to the introduction of antibiotic treatment the relationship between the community-level prevalence of TF in children correlated well with that of infection. The prevalence of TF was usually slightly greater than that of infection. Therefore, over a wide prevalence range, the community-level prevalence of TF in children is a reliable indicator that broadly reflects the underlying population burden of infection. The association between disease and infection for all ages was much weaker than that for children only; this supports the rationale for measuring TF in 1–9 year olds as the key indicator group for determining the need of antibiotic. The prevalence of TI was less well correlated than TF with infection before MDA, with the disease prevalence generally underestimating the level of infection.

At the individual level the utility of TF as a marker for infection is highly sensitive to the underlying prevalence of TF. Both the sensitivity and PPV rise substantially with increasing TF prevalence, and the specificity and NPV both drop. This sensitivity increase is therefore offset by a corresponding decrease in the specificity of this test in high TF prevalence communities. The usefulness of TF as an indicator of an individual’s infection status is dependent on both the sensitivity and the specificity of the test; too low a sensitivity leads to *C*. *trachomatis* infected individuals not receiving treatment, whereas too low a specificity leads to wasted resources treating uninfected individuals. The PPV possibly gives the clearest indication of where TF is useful as an indicator of an individual’s infection status; when the prevalence of TF is low (~5–10%), TF will only indicate an estimated 10% probability that the individual has *C*. *trachomatis* infection. This probability increases steadily with prevalence and once the community prevalence reaches 30%, the estimated PPV is in the order of 50–70%. Thus, where the population TF prevalence is above 30%, a positive TF diagnosis gives a 50–70% probability that an individual will be *C*. *trachomatis* infected.

After the introduction of MDA the relationship between the community prevalence of TF and chlamydial infection is less certain. Although the relationship between Active Trachoma and *C*. *trachomatis* infection appears to remain strong at the individual level, the population-level data suggests that post-MDA, Active Trachoma has a greater tendency to overestimate the underlying population prevalence of *C*. *trachomatis* infection. Below the 10% TF level the prevalence of infection was consistently low, and therefore below this level, TF prevalence appears to be a good marker for infection having being brought under control. However, in the studies where the prevalence of TF was above 10% after the introduction of MDA, the underlying prevalence of *C*. *trachomatis* infection was much less predictable. In some settings TF prevalence persists at high levels despite relatively little infection being detected. There might be several reasons for this observation. For example, if the loads of infection are substantially lower following the introduction of MDA these may not be so readily detected by diagnostic tests. Other bacterial species might also provoke a follicular conjunctivitis more readily in individuals previously infected with *C*. *trachomatis* [26]. However, whatever the explanation, it is likely that in some settings, the prevalence of TF will underestimate the impact MDA has had on the prevalence of *C*. *trachomatis*. This might lead to the on-going use of MDA after the infection has been adequately controlled.

There is much less published data on the relationship between TI and infection following the introduction of MDA. In general, the prevalence of TI appears to reduce more readily than TF. A number of studies have investigated the relationship between the load of infection and disease signs. These suggest that TI is particularly associated with high loads of infection [27]. Therefore, the decline in TI may reflect a shift to less intense infections. However, there were a few studies in which the prevalence of TI was low, but the prevalence of infection remained substantial.

We identified a reasonable number of studies reporting the community-level relationship between disease and infection prior to treatment, over a wide TF prevalence range. There were, however, fewer studies documenting the situation following the introduction of MDA, potentially limiting the conclusions that can be drawn after MDA. However, there was generally less detail in these reports about the individual-level relationship between clinical signs and infection. The studies came mostly from several East and West African countries, providing reasonable geographical coverage of the regions with the greatest trachoma burden. Standard WHO definitions of disease were generally used and the large majority of studies used the same commercially produced PCR assay, providing consistency across studies. However, there was some methodological heterogeneity. The age groups reported varied; where possible we use the data for children only in the meta-analysis to try to provide greater consistency between studies. The sample sizes varied considerably (from 56 to 7817); we weighted our analyses to adjust for size. The sampling methodology also varied considerably. The studies included were generally population-based samples or surveys of the entire resident population of a defined area.

Overall, the use of TF prevalence to guide the decision to initiate MDA in previously untreated districts appears to be reliable. In contrast, the situation following treatment is more uncertain, calling into question the reliability of clinical signs for monitoring progress towards the achievement of the elimination targets [28]. There are reports from hyperendemic regions that have received many rounds of high-coverage MDA that suggest that the prevalence of TF can be recalcitrant, even when *C*. *trachomatis* infection appears to have been brought under control. Therefore, contextually appropriate, cost-effective tests for *C*. *trachomatis* infection that can be administered in low-resource settings, and used to estimate the infection prevalence in a population-based sample, would be very helpful in guiding decisions around the cessation of MDA. Such tests are anticipated to reduce the number of annual rounds of MDA required and lead to the confirmation of trachoma control at an earlier stage.

## Supporting Information

S1 Checklist PRISMA Checklist (DOC)

S1 Fig PRISMA Flow Diagram (DOC)
