# Supplementary material for: The Relationship between Active Trachoma and Ocular Chlamydia trachomatis Infection before and after Mass Antibiotic Treatment
Source: PLoS Negl Trop Dis. 2016 Oct 26;10(10):e0005080. doi: 10.1371/journal.pntd.0005080 (PMC5082620; doi:10.1371/journal.pntd.0005080)
Supplement: S1 Fig — (DOC) [file pntd.0005080.s002.doc]

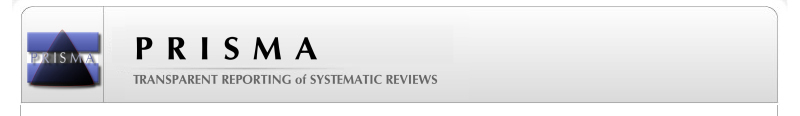
**PRISMA 2009 Flow Diagram**

**Screening**

**Included**

**Eligibility**

**Identification**

Records identified through database searching
(n = 718 )

Additional records identified through other sources
(n = 0 )

Records after duplicates removed
(n =718 )

Records screened
(n = 718 )

Records excluded
(n = 623)

Full-text articles assessed for eligibility
(n =95)

Full-text articles excluded, with reasons
(n = 33 )

Studies included in qualitative synthesis
(n =62 )

Studies included in quantitative synthesis (meta-analysis)
(n = 62)
